# PROSPECTIVE STUDY OF ASPIRIN FOR THROMBOEMBOLISM PROPHYLAXIS IN TOTAL HIP ARTHROPLASTY

**DOI:** 10.1590/1413-785220182602187265

**Published:** 2018

**Authors:** RAUL CARNEIRO LINS, EPITÁCIO LEITE ROLIM, FERNANDO DE SANTA CRUZ OLIVEIRA, SAULO MONTEIRO DOS SANTOS, TALE LUCAS VIEIRA ROLIM, FLÁVIO KREIMER

**Affiliations:** 1. Trauma and Orthopedics Service, Hospital das Clínicas, Department of Surgery, Universidade Federal de Pernambuco, Recife, PE, Brazil.; 2. Orthopedics Service, Hospital Otávio de Freitas, Recife, PE, Brazil.

**Keywords:** Arthroplasty, replacement, hip, Aspirin, Venous thrombosis/prevention & control, Artroplastia de quadril, Aspirina, Trombose venosa/prevenção & controle

## Abstract

**Objectives:**

To evaluate the effectiveness of aspirin as prophylaxis for deep venous thrombosis (DVT) in patients undergoing total hip arthroplasty (THA), and to analyze the incidence of bleeding during the post-operative period.

**Methods:**

This prospective study carried out in 2017 consisted of 37 patients indicated for THA with high risk for DVT. Immediately after the procedure, aspirin, elastic compression socks and early deambulation were initiated. Doppler ultrasound was performed in the legs 6 days and 6 weeks post-procedure to rule out venous thromboembolism. Hematometric variables and clinical criteria were used to detect bleeding.

**Results:**

The incidence of VTE (venous thromboembolism) 6 days post-procedure was 21.6%. By 6 weeks post-procedure, it dropped to 8.1%, (p = 0.102). Only 2.7% were diagnosed with VTE, 6 days and also 6 weeks post-procedure. Within the immediate postoperative period, hemoglobin was lower (p < 0.001), in contrast to 6 weeks after surgery, when it returned to baseline levels.

**Conclusion:**

Aspirin was an effective chemical prophylaxis for venous thromboembolism in high-risk patients who underwent THA. There was no clinical record of postoperative bleeding and hematometric levels suggested that there was no chronic bleeding. Level of Evidence II; Prospective study.

## RESUMO

**Objetivo:**

Avaliar a eficácia da aspirina como profilaxia da trombose venosa profunda (TVP) em pacientes submetidos à artroplastia total de quadril (ATQ) e analisar a incidência de possíveis sangramentos no pós-operatório.

**Método:**

Este estudo prospectivo realizado em 2017 foi constituído por 37 pacientes, com indicação de ATQ, com alto risco de TVP. No pós-operatório imediato foram iniciadas administração de aspirina, uso de meias de compressão elástica e deambulação precoce. Realizou-se um ultrassom com dopplerfluxometria no 6º dia e na 6ª semana de pós*-*operatório para excluir tromboembolismo venoso. Para detectar sangramentos, foram usadas variáveis hematimétricas e critérios clínicos.

**Resultados:**

No 6^o^ dia após o procedimento, a incidência de TEV (tromboembolismo venoso) foi 21,6% e na 6ª semana houve uma redução para 8,1% (p = 0,102). Apenas 2,7% foram diagnosticados com TVP tanto no 6^o^ dia como na 6ª semana. No pós-operatório imediato houve uma redução da hemoglobina (p < 0,001), contrastando com a sexta semana de pós-operatório, quando esses níveis se elevaram para os níveis basais.

**Conclusões:**

A aspirina mostrou-se eficaz como profilaxia química da trombose venosa em pacientes de alto risco submetidos à ATQ. Não houve registro clínico de sangramento pós-operatório e os níveis hematimétricos sugeriram não ter havido sangramento crônico. Nível de Evidência II; Estudo prospectivo.

## INTRODUCTION

Total Hip Arthroplasty (THA) has become a standard treatment for patients with high level degenerative arthritis, providing improvements in these patients quality of life. It is estimated that, in the USA, 350.000 THA are performed every year.^1^ ^,^ ^2^ In the UK, aproximately 70.000 surgeries of articular reconstruction using prosthesis are performed^3^ and, in Brazil, a national data registry of the usage of prosthesis is being implemented in order to provide more concrete data regarding these surgeries.^4^ ^,^ ^5^

Among the most feared complications of the arthroplasty are the thromboembolic diseases (TED), such as deep venous thrombosis (DVT) and the pulmonar thromboembolism (PTE). Prior to the routine administration of prophylaxis to TED, the incidence varied from 40% to 60% of distal asymptomatic DVT (below the popliteal vein), 15% to 20% of proximal lower limbs thrombosis, and from 0,5% to 2% of PTE.^1^ ^,^ ^6^ ^-^ ^14^ After the insertion of the utilization of prophylatic drugs, such as low molecular weight heparina, vitamin K inhibitors and the brand new Xa factor inhibitors, a significant reduction in the distal asymptomatic DVT incidence was observed, decreasing to about 20%, and the symptomatics to 1-3%.^3^ ^,^ ^15^ ^,^ ^16^

On the other hand, the use of these medicines have led to an increased incidence of coagulation disturbs, with gastrointestinal or surgical site bleedings.^17^ ^-^ ^19^ The routine administration of TED prophylaxis in patients undergoing THA is a consensus. However, until the present moment, neither the class of the drugs nor the time of use are well established for this matter.^1^ ^-^ ^6^ ^,^ ^16^ ^,^ ^20^ The lack of multicentric studies, variety of protocols and different DTE risk factors among the patients make it difficult to standardize a ideal prophylaxis.^16^ ^,^ ^19^

The *American College of Chest Physicians* (ACCP), in their last update, in 2012, approved, for the first time, the isolated use of aspirin as TED prophylaxis in patients undergoing THA.^15^ ^,^ ^17^ ^-^ ^19^ ^,^ ^21^ Among the advantages of aspirin there are the low cost, the easy access by the population in the public health system and the nonnecessity of keeping the patients hospitalized to monitor any coagulation disturbs that may occur, as in the use of warfarin.^9^ ^,^ ^20^ Just like the ACCP, the *American Academy of Orthopaedic Surgeons* (AAOS) authorize the use of aspirin and recommends the mechanical prophylaxis associated with early deambulation for these patients.^8^ ^,^ ^9^ ^,^ ^11^ ^,^ ^20^

This study has the objetive to evaluate the efficiency of the isolated use of aspirin as prophylatic therapy in patients undergoing THA (high risk for TED), associated with early deambulation and elastic compression socks. 

## MATERIALS AND METHODS

Analytical prospective study carried out during the period bettween March and June of 2017, where 40 patients with cardiovascular risk among 1 and 2, based on Goleman criteria^22^, were selected to realize THA. One patient were excluded because of an intraoperatory complication (acetabular fracture), and the other two were excluded because of non adhesion of the proposed prophylatic scheme. Provide that, we studied 37 patients with indication of THA, all of them with high TED risk, based on Caiafa and Bastos^23^ criteria. The prophylatic scheme used consists of aspirin 650 mg per day, divided in two takes of 325 mg, with a 12 hours interval bettween them, during 30 days. The patients, after surgery, started the using of elastic compression socks, still in the post-anesthetic recovery room, and then they were oriented to deambulate, as soon as possible, already in the first post operative day. The selection of patients were determined by the Goldman criteria - reaching those characterized with cardiovascular risk bettween 1 and 2 - and by the Caiafa and Bastos criteria, including only those considered with high risk for TED^23^. We excluded patients with 3 or 4 goldman´s criteria, patients in previous use of anticoagulant drugs, recent gastrointestinal bleeding episodes, previous hematological diseases and revision surgeries. The risk factors for TED are presented in Chart 1. 

**Chart 1:** Evaluation of DVT risk .

PROPOSED ASSESSMENT OF DVT RISK			
Protocol Screen			
1. Risk factors			
▪ General anesthesia	▪ COPD*	▪ Regional Ileitis	▪ Obesity
▪ Oral contraceptives	▪ Eclampsia	▪ Prolonged confinement to hospital bed (>3 days)	▪ IM paralysis
▪ Cancer*	▪ Large burns	▪ Limb immobilization	▪ Pre-eclampsia
▪ Long-term central venous catheter	▪ CHF	▪ Heart attack	▪ Puerperal
▪ Prolonged surgery	▪ Age >40 years	▪ Severe infection	▪ Chemotherapy
▪ Autoimmune disease	▪ Age >60 years*	▪ ICU hospitalization	▪ Ulcerative colitis
▪ Nephritic syndrome	▪ Trauma	▪ Severe trauma*	▪ HRT
▪ Large-diameter varicose veins	▪ None	▪ Other	
2. The following patients are considered as high risk of VTE			
▪ Major orthopedic surgeries of the hip/knee	▪ Stroke	▪ Transvesical prostatectomy	
▪ Major surgeries for cancer	▪ Thrombophilia		
▪ Spinal trauma	▪ Past history of DVT/PE		
3. Risk classification			
▪ Low (0 to 1 point)	▪ Medium (2 to 4 points)	▪ High (5 or more points)	

The patients underwent to THA, under raquidian anesthesia, by the posterior lateral access, with the patients positioned in contralateral decubitus in relation to the operated side. After surgery, still in the post-anesthesia recovery room, the patients dressed the elastic compression socks, and after 12 hours aspirin was initiated, 650 mg divided in two takes of 325 mg, during 30 days. The deambulation were started and supervisioned by the physiotherapy team in the first post-op day, taking into consideration the pain as a potential limitant factor of the march. The criteria adopted to diagnose post operative bleedings were the ones recommended by the National Institute for Health and Care Excellence (NICE), which are patient death, more than 2 points decrease in hemoglobina, hemotransfusion of more than two units of red blood cells concentrates, necessity of surgical reassessment because of surgical site hematoma or retroperitoneal, cranial or thoracic bleeding.^24^ ^,^ ^25^

 In the 6th post-operative day the patients underwent to a venous doppler ultrasound to check low deep venous thrombosis (below the popliteal vein), and they were examined to verify any signs of thromboembolic disease. The ultrasound exams were conducted by two radiologists with experience in ultrasonography with dopplerfluxometry, and every identified case of TED were confirmed by both of them. 

Considering the períod of maximum incidence of TED in orthopaedic surgeries bettween the 6th and the 28th day after surgery,^15^ ^,^ ^25^ the patients were submitted to another ultrasound exam, at the 6th week after surgery, by the same radiologists, using the same protocol, and they were interrogated regarding gastrointestinal or surgical site bleedings that may have occured. They have also collected blood samples to analyze hemoglobin and hematocrit levels.

To diagnose TED, a qualitative variable were used: presence of venous clots below the popliteal vein, in only one or both lower limbs, symptomatics or not, confirmed by the two radiologists of the research team. On the other hand, to detect possible bleedings, caused by the aspirin, we took into consideration hematological quantitative variables: hematocrit and hemoglobin in the pre-operative, immediate post-operative and late post-operative period (6th week), besides the physical examination and the clinical history of gastrointestinal or surgical site bleeding referred by the patient.

This research Project was submitted for approval by the ethics committee in research with human beings of the *Centro de Ciências da Saúde (CCS)* of UFPE, and it was protocoled under the CAAE n^o^ 66155517.2.0000.5208. The obtaining of the free and informed consent term was realized by the main researcher, including the steps contained in the document, following the orientations of the 466/12 resolution of the Health Ministry.

To analyze the data, we created a spreadsheet at Microsoft Excel, which was moved to a SPSS software, 18 version, where the analysis took place. To evaluate the demographic profile of the patients in this study, percentage frequencies of the studied variables were calculated and the frequency distributions were determined. For the quantitative variables, we calculated the following statistics: minimum, maximum, mean and standard deviation. The Chi-Square Test was applyed to compare the percentages that were found in the independente variables. 

The normality of the hemoglobin and hematocrit levels in the patients blood were evaluated by the Shapiro-Wilk Test, and, in those cases where the normality was indicated, we applied the Student Test for paired samples to compare the mean of hematocrit and hemoglobin levels relating to the pre-operative period, immediate post-operative period and late post-operative (6th week after surgery).

When comparing the prevalences of clots, we created a contingency table and applied the Exact Fisher Test. All of the conclusions were made taking into consideration the significance level of 5%.

## RESULTS

The Table 1 presents the distribution regarding age and gender of the patients. The age varied form 18 to 71 with mean ± standard deviation (49,2 ±14,7). The majority is female19 (51,4%) with age up to 59 years old, mean of 26 (70,3%). The proportion comparation test was significant for the age factor (p-value= 0,014), indicating that the proportion of genders are similar. However, the percentage for each age group studied wasn’t significant.

**Table 1:** Patients distribution based on gender and age.

Factor	n	%	p-value¹
Gender			
Male	18	48,6	0,869
Female	19	51,4	
Age			
≤ 59	26	70,3	0,014
≥60	11	29,7	
Minimum-Maximum	18-71		-
Mean ± Standard deviation	49,2 ± 14,7		-

¹p-valor for Chi-squared test to compare proportions (if p-value< 0.05, the level percentages for the evaluated characteristic differ significantly).

The Table 2 presents the prevalence of clots in the 6th post-operative day and at the 6th week after surgery. It was verified that in the 6th day there was a bigger prevalence of VTE (21,6%), despite there was a reduction at the 6th week to 8,1%. Although, the homogeneity test wasn’t significant (p-value¹ = 0,102), indicating that the distribution of VTE in the 6th day and at the 6th week were similar. Furthermore, it was observed that, from the totality of patients, only 2,6% were diagnosed with DVT in both, the 6th day and at the 6th week after surgery. 

**Table 2:** Prevalence of clots based on the studied period.

Factor	Period		p-value¹
	6^th^ day	6^th^ week	
VTE			
Yes	8(21,6%)	3(8,1%)	0,102
No	29(78,4%)	34(94,9%)	
DVT			
Yes	1(2,7%)	1(2,7%)	1,000
No	36(97,3%)	36(97,3%)	

**Figure 1:**
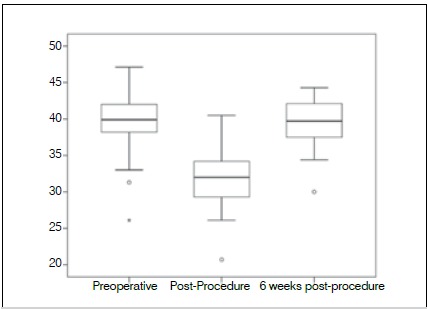
Box plot showing hematocrit level at the three assessment times.

The Table 3 shows the descriptive analysis of hematocrit and hemoglobins levels based on the period of evaluation. It is observed that the hematocrit mean suffered a significant reduction bettween the pre and pos-op periods (mean of 39,81 in the pre-operative period decreases to 31,86 in the immediate post-operative period p-value < 0,001). On the other hand, at the 6th post-op week, there was a significant increment in the hematocrit mean (mean = 39,39, p-value < 0,001), getting statistically close to the pre-operative period numbers (p-value = 0,476). (Figure 1)

**Table 3:** Descriptive analysis of hematocrit and hemoglobin leves bettween the pre-op, immediate post-op and late (6th week after surgery) post-operative periods.

Mean	Evaluation period		
	Pre-op	Immediate post-op	Late post-op
Hematocrit (HT)	39,81^a^ ± 4,30	31,86 ± 3,93	39,39^a^ ± 3,40
Hemoglobin (HB)	13,81 ± 1,38	11,10 ± 1,36	13,17 ± 1,29

**Figure 2:**
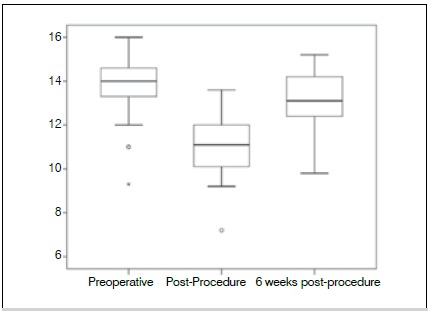
Box plot showing hemoglobin level at the three assessment times.

In relation to hemoglobin, there was a significant reduction in the immediate post-op (mean of 13,81 in the pre-op, and 11,10 in the immediate post-op, p-value <0,001), although, at the 6th post-op week, the hemoglobin levels raised (mean = 13,17, p-value = 0,001), but it remains statistically lower than the levels at the beginning of the treatment (p-value < 0,001). (Figure 2) 

In the Table 4 we registered the distribution of the occurence of clots in the immediate post-op and at the 6th week. From the total, 8 patients (21,6%) presented clots in the immediate post-op, while only 3 cases (8,1%) were identified at the 6th post-op week. It was observed that the highest incidence of clots in the post-operative period occured in patients who were operated bilaterally (25%), when compared with the group that had only a one side surgery (21,7%). However the independency test wasn’t significant (p-value= 1,00), demonstrating that the prevalence of clots in both groups are similar.

**Table 4:** Distribution of clots occurence in the 6th day and at the 6th week of the post-operative period.

Bilaterality	Clots 6^th^ day		Clots 6th week	
	Yes	No	Yes	No
No	6(21,7%)	23(78,3%)	2(6,9%)	27(93,1%)
Yes	2(25,0%)	6(75,0%)	1(12,5%)	7(87,5%)
*p*-value¹	1,000		0,530	

¹P-value for Fisher’s exact test (if p-value < 0.05 the prevalence of clots differs bilaterally and unilaterally among groups).

There wasn’t any episodes of surgical site bleeding, nor even any bleeding signs according to the NICE criteria.

## DISCUSSION

The non-standardization of multicentric studies, the heterogeneity of the studied groups and the variety of options for chemical and mechanical prophylaxis are some of the factors that collaborate to the absence of a consensus regarding the ideal and universal prophylaxis for patients undergoing total hip arthroplasty. ^1-3^

Chari et al.,^26^ in 2012, presented a meta-analysis study, comparing several protocols that exists in different countries, and concluded that there isn’t a consensus about the ideal method for TED prophylaxis in patients undergoing THA, with different levels of recommendation regarding the chemical agentes, period of use and clinical criteria. 

Aiming to turn the sample of our study more homogeneous, we selected patients with cardiovascular risks bettween 1 and 2, based on Goldman criteria,^22^ and, according to the Caiafa and Bastos criteria^23^, published in 2001, all of them were considered high risk for thromboembolic diseases. Besides, none of the selected patients presented previous history of neither gastrointestinal bleeding nor use of anticoagulants.

The use of aspirin as chemical prophylaxis for thromboembolic events in patients undergoing total hip arthroplasty is being reported as successful since the decade of 1990. McCardel et al^27^ published, in 1990, a prospective work, using aspirin as prophylaxis and a Doppler ultrasound for screening of clots in lower limbs. They found an incidence of deep venous thrombosis of 5,7% in 159 patients submitted to THA. In our study, with a similar methodology, we found 2,7% of deep venous thrombosis in the 6th post-operative day and, also, at the 6th week.

 Ibrahim et al.,^28^ analyzed retropectively more than 28 thousand patients submitted to THA, comparing the use of aspirin and warfarin. They concluded that both drugs were equivalent in the VTE prophylaxis, but the aspirin presented a lower incidence of bleeding related complications than the warfarin. In our study, there wasn’t any registry of bleeding related complications - such as gastrointestinal or surgical site bleeding - and, after 6 weeks of the surgery, hematocrit and hemoglobin levels have returned to the pre-operative levels.

An et al^20^ published, in 2015, a meta-analysis about the prophylatic use of aspirin during the post-operative period of arthroplasty. They found a DVT incidence of 1,2%, and 0,3% of bleeding. In the present study, we found a DVT incidence of 2,6% in both the 6th day and at the 6th week (one case). Considering the incidence of VTE in the 6th post-op day, we identified, by a Doppler Ultrasound, 8 (21,6%) cases, and, at the 6th week, only 3 (8,1%) cases were diagnosed, showing the resolution of five of the previously identified clots.

We are aware that the small population of the study and the lack of a group control are limitant factors of our study. However, we worked to turn the group the most homogeneous possible in terms of variables related to the epidemiological profile of the patients. The lack of bleeding episodes confers safety to the use of aspirin as a prophylatic drug in the post-operative period of total hip artrhoplasty.

## CONCLUSIONS

 Our study recommends the use of aspirin as the chemical prophylatic agent for venous thromboembolism in patients undergoing total hip arthroplasty, associated with the use of mechanical prophylaxis and early deambulation - started in the first 24 hours after surgery (immediate pos-operative period). 
